# Combined Diabetes Education/Skills Training and Social Needs Resolution Intervention for Older African Americans with Poorly Controlled Type 2 Diabetes (DM Social Needs): Study Protocol for a Randomized Controlled Trial

**DOI:** 10.3390/healthcare12191991

**Published:** 2024-10-06

**Authors:** Aprill Z. Dawson, Rebekah J. Walker, Tatiana M. Davidson, Rebecca Knapp, Leonard E. Egede

**Affiliations:** 1 Division of General Internal Medicine, Department of Medicine, Medical College of Wisconsin, 8701 Watertown Plank Rd, Milwaukee, WI 53226, USA; 2 Center for Advancing Population Science, Medical College of Wisconsin, 8701 Watertown Plank Rd, Milwaukee, WI 53226, USA; 3 Division of Population Health, Department of Medicine, Jacobs School of Medicine & Biomedical Sciences, University at Buffalo, 77 Goodell Ave., Buffalo, NY 14203, USA; rebwalker@mcw.edu (R.J.W.); legede@mcw.edu (L.E.E.); 4 Department of Nursing, College of Nursing, Medical University of South Carolina, 96 Jonathan Lucas Street, Charleston, SC 29425, USA; davidst@musc.edu (T.M.D.);

**Keywords:** older adults, type 2 diabetes, randomized controlled trial, behavioral intervention, social needs, diabetes self-management education, hemoglobin A1C, blood pressure, cholesterol

## Abstract

Background: Approximately 11.3% of the US population has diabetes. The burden of diabetes is higher in older adults and African Americans (AAs), such that 40% of adults aged 50 years and older have diabetes; African Americans are 60% more likely to be diagnosed with diabetes compared to non-Hispanic Whites (NHWs). Structural racism has resulted in fewer economic and educational opportunities for AAs, higher social risks, and unmet basic needs, which result in financial instability, housing instability, food insecurity, and a lack of transportation compared to NHWs. The presence of these unmet basic needs is a driver of poor adherence to diabetes self-management in older AAs. Aim: To test the preliminary efficacy of a nurse case-manager, telephone-delivered intervention that provides foundational diabetes self-management education and skills training, while also addressing and resolving the unmet social needs of older AAs with poorly controlled type 2 diabetes mellitus (T2DM). The primary hypothesis is that older AAs with T2DM randomized to the DM Social Needs intervention will have significantly lower hemoglobin A1C (HbA1C), blood pressure, and LDL levels compared to the usual care arm at 6 months post randomization. Methods: This is a three-year prospective, randomized clinical trial that will enroll 100 AAs aged 50 and older with type 2 diabetes (T2DM) and HbA1C ≥ 8% into one of the following two groups: (1) a nurse case-manager, telephone-delivered intervention that provides foundational diabetes self-management education and skills training, but also addresses and resolves unmet social needs; or (2) an enhanced usual care group that will receive mailed diabetes education materials. Participants will be followed for 6 months to determine the effect of the intervention on HbA1C, blood pressure, and low-density lipoprotein (LDL) cholesterol levels. Results: Baseline characteristics will be presented by study group, and within- and between-group changes in primary outcomes from baseline to 6 months will be reported. Conclusion: The results from this study will provide insights into the efficacy of a combined diabetes education and skills training and social needs resolution intervention for older African Americans with poorly controlled type 2 diabetes and will inform strategies to improve diabetes outcomes for this vulnerable population.

## 1. Introduction

Approximately 11.3% of the US population has diabetes and the annual cost of diabetes is about USD 327 billion [1]. More than 50% of the lifetime medical cost of diabetes is due to diabetes-related complications [2]. Individuals with diabetes are twice as likely to have microvascular (retinopathy, nephropathy, and neuropathy) and macrovascular (coronary heart disease, cerebrovascular disease, and peripheral vascular disease) complications compared to those without diabetes [3,4,5,6]. The burden of diabetes is higher in older adults, such that 40% of adults aged 50 years and older have diabetes, over 60% of diabetes costs are attributed to Medicare and Medicaid, and older adults experience more complications compared to younger adults [4,7,8]. As the US population continues to age, the prevalence of diabetes will continue to rise in older adults [8,9,10,11]. 

The prevalence of diabetes is higher in African Americans (AAs) and they are 60% more likely to be diagnosed with diabetes compared to non-Hispanic Whites (NHWs) [12]. On average, AAs are diagnosed with diabetes at a younger age [13], are more likely to experience complications, are four times more likely to have an amputation, and are twice as likely to die as a result of diabetes compared to NHWs [12,13,14,15,16]. The higher rate of complications in AAs is due to the less optimal control of hemoglobin A1C (HbA1C), blood pressure, and low-density lipoprotein (LDL) levels compared to NHWs [12,13,14,15,16,17]. Although optimal diabetes control can be achieved by adherence to diabetes self-management guidelines including medication adherence, blood glucose self-monitoring, attending doctor’s visits, healthy diet, and exercise, AAs are less adherent to diabetes self-management guidelines compared to NHWs [18].

Structural racism and the reinforcement of inequitable systems over time has resulted in fewer economic and educational opportunities for AAs, higher social risks, and unmet basic needs, resulting in financial instability, housing instability, food insecurity, and lack of transportation compared to NHWs [16,19]. For example, the proportion of older AAs living in poverty is higher than that of NHWs (19% vs. 7%); food and housing insecurity levels are higher in AAs compared to NHWs (44% vs. 22%); and AAs have eight times less wealth compared to NHWs [20,21,22,23,24]. The presence of these unmet basic needs is a driver of poor adherence to diabetes self-management in older AAs, including medication adherence, glucose self-monitoring, healthy eating, completing medical visits, and purchasing necessary diabetes care supplies [18,25,26,27,28]. 

However, there is evidence that basic needs referral is not sufficient to address unmet social needs [29]; therefore, to achieve optimal outcomes for older AAs with type 2 diabetes mellitus (T2DM) and unmet social needs, it is imperative to combine traditional diabetes education and skills training with strategies to identify and resolve unmet social needs. Therefore, to address this gap in knowledge, we will use a randomized controlled trial design to test the preliminary efficacy of a combined diabetes education/skills training and social needs resolution (DM Social Needs) intervention on improving clinical outcomes for older AAs with T2DM. The principal investigator (PI) (AZD) and research team are well suited to complete this study. The team has been successful in previously recruiting, enrolling, and completing clinical trials with older African American patients with poorly controlled type 2 diabetes [30,31,32,33,34]. 

### Study Aims and Objectives

The primary aim of this study is to test the preliminary efficacy of the DM Social Needs intervention on clinical outcomes (HbA1C, blood pressure, and LDL levels) in older AAs with uncontrolled T2DM. The secondary aim is to test the preliminary efficacy of the DM Social Needs intervention on patient-reported outcomes (based on PROMIS measures) in older AAs with uncontrolled T2DM. The primary outcome is HbA1C at 6 months post randomization, the secondary outcome is blood pressure at 6 months post randomization, and the tertiary outcomes are LDL and patient-reported outcomes of global health, fatigue, pain interference, and social participation at 6 months post randomization. 

## 2. Methods

This three-year randomized clinical trial will test the efficacy of the six-session nurse case-manager, telephone-delivered combined diabetes education/skills training and social needs resolution (DM Social Needs) intervention on improving clinical outcomes for AAs aged 50 and older with poorly controlled type 2 diabetes (HbA1C ≥ 8%). Funding was awarded in November 2022 and has an anticipated end date of November 2025. One-to-one individual-level randomization will be used to assign eligible participants to study groups. Outcome assessments will occur at baseline, 3 months, and 6 months post randomization.

### 2.1. Ethics and Trial Registration

This study is funded by a grant from the American Diabetes Association (ADA) (11-22-JDFHD-01; PI: Dawson). The trial and study protocol were approved by the Medical College of Wisconsin Institutional Review Board on 24 May 2023 (PRO00046456). The trial was registered on ClinicalTrials.gov (trial number: NCT05935410) on 27 June 2023. 

### 2.2. Trial Status

This study was funded in November 2022 and was assigned protocol number PRO00046456. As of September 2024, 53 of the 100 total participants have been randomized. Recruitment is expected to be completed by May 2025.

### 2.3. Trial Population and Recruitment

A total of 100 AAs aged 50 and older with poorly controlled T2DM (HbA1C ≥ 8%) will be randomized in a 1:1 ratio into one of two study groups—(1) nurse-led, telephone-delivered DM Social Needs intervention that includes education and skills training on diabetes management and problem solving and the resolution of social needs during monthly, 60 minute phone calls (*n* = 50); and (2) an enhanced usual care group that will receive mailed diabetes education materials each month for 6 months in accordance with recommendations from the ADA (*n* = 50). Participants will be followed for 6 months to evaluate the preliminary efficacy of the intervention on clinical outcomes (HbA1C, blood pressure, and LDL levels) and patient-reported outcomes (PROs).

A multifaceted approach will be used to recruit AA older adults with poorly controlled T2DM after receiving approval from the Institutional Review Board (IRB). (1) Housing Authorities. The study PI will meet with housing authority leadership for public and senior housing communities to obtain approval to recruit and share information about the study at their locations. (2) Community Leaders. The study PI will meet with other community leaders including church/religious leaders, small business owners, and federally qualified health center and community center leaders to obtain approval to recruit and share information about the study at their locations. (3) Mailing Lists. Targeted mailing lists obtained from electronic health records and other publicly available sources will be used to send letters describing the study and providing instructions on how to contact the research team will be sent to older African Americans in an effort to reach eligible patients. (4) Referrals. Referrals will be accepted from healthcare providers, community members, or other study participants. Individuals who are referred to participate will be contacted by the research team to assess interest, willingness, and eligibility to participate.

Individuals who are interested in participating will be scheduled for screening. Research staff will obtain signed informed consent prior to initiating study procedures for all participants. Study eligibility criteria are as follows: Inclusion: (1) age 50 and older; (2) self-identified as AA or Black; (3) self-reported diagnosis of T2DM; (4) HbA1C ≥ 8% at the screening visit. Exclusion: (1) self-reported participation in other diabetes clinical trials; (2) alcohol or drug abuse or dependency, as assessed by the CAGE-AID; (3) mental confusion at screening assessment, suggesting significant dementia; (4) life expectancy < 6 months. All treatments including insulin, oral medications, both, or neither were acceptable for the study, and eligible participants were enrolled regardless of treatment. 

### 2.4. Randomization

A permuted block randomization method will be used to assign patients to control or intervention. Block size will be varied to minimize the likelihood that the blind will be broken. The randomization will be computer generated and stratified by screening HbA1C levels (8–10% vs. >10%). Study staff responsible for completing surveys and collecting measures at baseline, 3 months, and 6 months post randomization will be blinded to study group assignment. 

### 2.5. Combined Diabetes Education/Skills Training and Social Needs Resolution (DM Social Needs) Intervention

The proposed outline of the six-session nurse-led, telephone-delivered DM Social Needs intervention includes education and skills training on diabetes management, problem solving, and resolution of social needs during monthly, 60 minute phone calls. Intervention sessions will be delivered by trained nurse case-managers using diabetes education and skills training materials in accordance with ADA recommendations and guidelines. The baseline session will include an intake using the accountable care organization social determinants of health screening tool [35] to identify and prioritize the patient’s top three social needs to resolve during the six-month study. Diabetes education and skills training topics will be discussed monthly and include an overview of diabetes; blood glucose monitoring; medication adherence; diet; exercise; blood pressure; cholesterol; and foot, skin, and dental care. Prioritized social needs will be addressed using existing community-based social services in order to resolve prioritized needs. 

### 2.6. Enhanced Usual Care Group

Participants in the usual care arm will receive mailed diabetes education materials in accordance with recommendations from the American Diabetes Association (ADA) monthly [36]. Diabetes education material will be based on ADA education documents that are publicly available and will cover the following topics: (1) overview of diabetes; (2) self-monitoring of blood glucose; (3) medication adherence; (4) basics of eating and exercise; (5) blood pressure and cholesterol; and (6) foot, skin, and dental care.

### 2.7. Study Instruments and Data Collection Schedule

**Figure 1:**
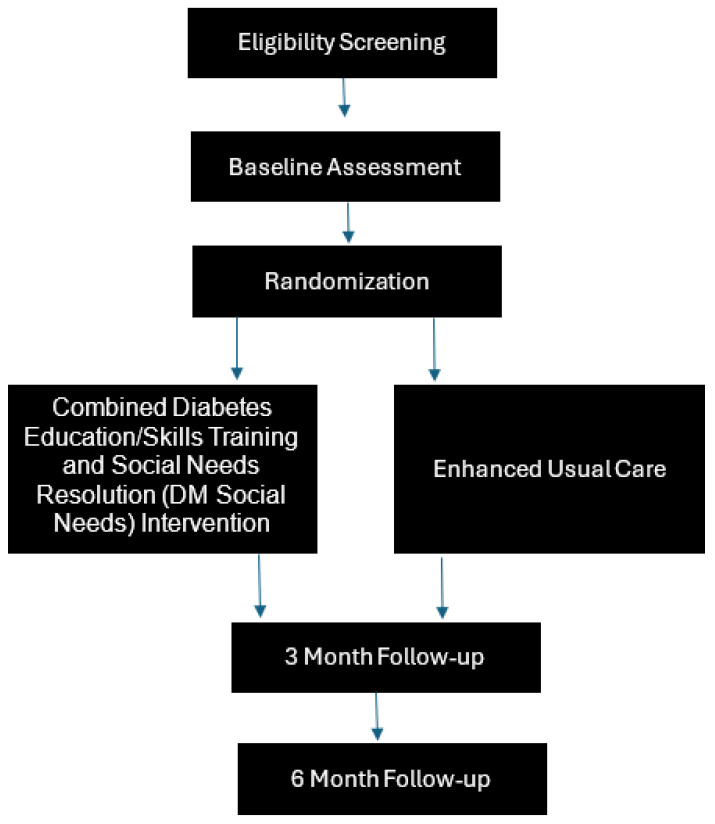
Flow diagram of study design.

Figure 1 is a flow diagram of the study design and visit schedule. Table 1 shows the data collection schedule, and Table 2 provides a description of the measures and scales used to collect data. 

### 2.8. Primary Outcomes

The three primary outcomes for this study are HbA1C, blood pressure, and low-density lipoprotein cholesterol levels. Primary outcomes will be collected at baseline, 3 months, and 6 months post randomization. 

### 2.9. Data Management

Research Electronic Data Capture (REDCap) is a secure, web-based application that will be used for data management. The PI will monitor data quality and integrity and will meet with co-investigators and the study team weekly to discuss recruitment, retention, and data entry. 

### 2.10. Sample Size and Power

Clinical outcomes (HbA1C, blood pressure, and LDL levels) and patient-reported outcomes (based on PROMIS measures) will be assessed at baseline, 3 months, and 6 months post randomization with the primary time point being at 6 months post randomization. Assuming two post randomization measurement time points, with a level of significance α = 0.10, and given that this is a pilot study [48], two-tailed comparison, the correlation between pairs of repeated measurements within participants (interclass correlation) no larger than ρ = 0.5, with an AR (1) covariance structure, we estimate that 40 patients per intervention group are needed to detect, with 80% power, a standardized effect size of 0.3 sd (Cohen’s d) for the quantitative outcomes for Aims 1–2. For example, for the outcomes HbA1C, systolic blood pressure, and LDL, assuming standard deviations (sds) of 1.9 percentage points, 19.9 mmHg, and 20 mg/DL, respectively, the corresponding raw effect sizes that can be detected between the intervention and control group are 0.57 percentage points, 6.0 mmHg, and 6.0 mg/DL, respectively. Intervention sample sizes of 40 patients per intervention group will produce 90% confidence interval (CI) estimates of effect sizes (differences in intervention means) with a precision of ±0.26 sds for study outcomes. To account for the missing information in the intent-to-treat (ITT) sample and the dilution effect of ITT analyses, we will increase the sample size by 20% to achieve a final ITT sample size of 50 patients randomized to each treatment group. Because the objective is to obtain preliminary evidence of efficacy rather than to definitively confirm/refute hypotheses, we have used less stringent criteria for power, level of significance, and confidence interval estimates (i.e., power = 0.8, alpha = 0.1, and 90%CI) than for a subsequent confirmatory randomized controlled trial (RCT). 

### 2.11. Data Analysis

The intent-to-treat (ITT) sample comprises all randomized participants. Univariate descriptive statistics and frequency distributions will be calculated for clinical and other baseline covariates for the total sample to describe the study population, and by intervention group to assess the similarity of the treatment arms at baseline to confirm randomization success and to identify potential confounding variables to be used as covariates in secondary analyses. We will use a longitudinal general linear model approach as the analytic framework for inferential analyses. This method allows for missing data under the assumption of missing at random (MAR), accounts for the correlated longitudinal measurements within subjects, and accommodates a wide range of distributional assumptions through the specification of appropriate link functions. For the primary analyses of patient clinical outcomes (HbA1C, blood pressure, and lipid levels) and patient-reported outcomes, the identify link function will be used to compare 6 month treatment means and longitudinal trajectories for the DM Social Needs and usual care arms. Each outcome within each domain will be used separately as the dependent variable in the models with intervention, time, and time-by-intervention as the primary independent variables and baseline score for a given outcome variable as an adjustment covariable. In further secondary analyses, covariates (e.g., sociodemographics, health literacy, and comorbidities) will be added to each model to adjust for confounding variables, if indicated. Model-generated least squares intervention means, and the intervention mean difference (effect size), for clinical and patient-reported outcomes will be estimated, and intervention differences will be compared for significance at the primary endpoint (6 month) using 90% confidence intervals and *p*-values for appropriate model-derived contrasts. 

## 3. Results

The study team has recruited 53 out of 100 participants to date, and is on track to complete the study prior to the end of the funding period. Data analyses will be conducted following the completion of enrollment and follow-up visits for all 100 participants. The results from this study will include the final number of total study participants along with the number of participants assigned to the DM Social Needs intervention and enhanced usual care groups. Additionally, a CONSORT diagram will be used to show the flow of patients through the study. Baseline characteristics for demographics for the ITT sample will be presented by study group. Within- and between-group changes in the three primary outcomes of HbA1C, blood pressure, and LDL levels from baseline to the 6 month primary endpoint will also be reported.

## 4. Discussion

This novel study will not only offer diabetes education and skills training to an older adult African American population with a high risk of poor diabetes-related outcomes, but it will also offer insights into understanding the impact of going beyond social needs referral and navigation to social needs resolution on clinical and self-reported outcomes. If it is established that resolving social needs and diabetes education and skills training are important in diabetes control; future studies will be planned to implement the DM Social Needs intervention at a population level, and efforts will be made to inform policy change to facilitate payors to use scalable efforts to improve outcomes for older African Americans with diabetes.

A number of strategies will be employed to address potential pitfalls and any high-risk aspects of the proposed work. First, potential pitfalls exist regarding the completion of the study, including recruitment and retention. The recruitment of 100 participants meeting the inclusion and exclusion criteria is not expected to pose a concern based on prior methods developed and used by the research team. The study team has successfully enrolled 53 participants to date and is on target to complete enrollment according to the planned timeline. Based on previous discussions with community partners, improving diabetes outcomes for older adults is a topic of concern and African American communities are interested in participating in studies to address this problem. While the retention of participants is of concern, strategies known to be effective based on prior R01 studies conducted by collaborators (LEE, RJW, and TMD) will be used. These strategies include providing appointment cards for follow-up visits, sending appointment reminders, and offering incentives at visits to compensate for inconvenience and time. Similarly, the research team has existing strategies in place for delivering the intervention without contamination, and ensuring fidelity to the protocol. Prior to initiating any study procedures, a protocol training will be held with the research team including research assistants and health educators that includes detailed discussion of the rationale for the study and the intervention. 

The anticipated duration of the study is three years. In the first quarter of Year 1, we will finalize the clinical trial protocol and register the study on clinicaltrials.gov, finalize consent forms and obtain IRB approval, develop standard operating procedures, and train study staff. We will randomize 30 patients by the end of Year 1 (30%), 70 patients by the end of Year 2 (70%), and 100 patients by Quarter 2 of Year 3 (100%); with an average of 4 patients enrolled per month. Each patient will be followed for 6 months with study visits at baseline, 3 months, and 6 months post randomization. The intervention will be delivered monthly for 6 months, and follow-up will take place during years 1–3. The completion of this project will provide preliminary data for an adequately powered problem solving and social needs resolution intervention by Year 3 of the award. This study will pave the way for the optimization of care delivery among older AAs with T2DM and social needs, thereby reducing complications, improving quality of life for older AAs with poorly controlled T2DM (Table 3). 

Study limitations include a relatively small sample size, as is the norm for pilot studies. Preliminary results generated after completing the study will be used to submit an NIH R01 proposal for a larger study that will be powered to assess the efficacy of the intervention on diabetes-related outcomes. In addition, by enrolling only African American adults aged 50 and older, study findings will not be generalizable to the general older adult population. 

## 5. Conclusions

Overall, the results from this study will provide insights into the efficacy of a combined diabetes education and skills training and social needs resolution intervention for older African Americans with poorly controlled type 2 diabetes and inform strategies to improve diabetes outcomes for this vulnerable population.

## Figures and Tables

**Table 1:** Data collection schedule.

Measures	Assessment		
	Baseline	3 Months	6 Months
Primary Outcomes			
Hemoglobin A1C	x	x	x
Blood pressure	x	x	x
Low-density lipoprotein cholesterol	x	x	x
Secondary Outcomes (PROMIS Measures)			
Global health	x	x	x
Fatigue	x	x	x
Pain	x	x	x
Social participation	x	x	x
Process and Mediators/Moderators			
Accountable Health Communities’ Health-Related Social Needs (i.e., housing instability, food insecurity, and transportation)	x	x	x
Summary of Diabetes Self-Care Activities (SDSCAs)	x	x	x
Medication adherence	x	x	x
Diabetes knowledge	x	x	x
Depression	x	x	x
Anxiety	x	x	x
Covariates			
Sociodemographics	x	x	x
Health literacy	x	x	x
Comorbidities	x	x	x

**Table 2:** Description of the measures and scales used to collect data.

Variable	Method Measured
Primary Outcomes	
Hemoglobin A1C	Blood will be collected and analyzed at baseline, 3 months, and 6 months.
Blood pressure	Blood pressure readings will be obtained using automated blood pressure monitors (OMRON IntelliSenseTM HEM-907XL, Dalian, China). Three readings will be collected and the average will be documented as the participant’s blood pressure.
LDL cholesterol	Blood will be collected and analyzed at baseline, 3 months, and 6 months.
Secondary Outcomes	
Global health	The global health PROMIS measure is a 10-item questionnaire to measure overall physical and mental health [37].
Fatigue	The short form 8a PROMIS measure is an 8-item questionnaire to assess how fatigued an individual felt during the last 7 days [38].
Pain interference	The 8-item pain interference PROMIS measure assesses how much pain interfered with activities during the last 7 days [39].
Social participation	The Ability to Participate in Social Roles and Activities scale is an 8-item measure that asks how often an individual has trouble with or has to limit participation in social activities [38].
Process and Mediators/Moderators	
Social needs	Ten questions from the Accountable Health Communities’ Health-Related Social Needs screening tool on the domains of housing, food insecurity, transportation, and utilities will be used to assess unmet social needs [35].
Diabetes knowledge	Diabetes knowledge will be assessed with the Starr County Knowledge Questionnaire
	(DKQ)—a 24-item assessment with a reliability coefficient of 0.78 [40].
Summary of Diabetes Self-Care Activities (SDSCAs)	This scale assesses diabetes self-care activities including general diet, specific diet,
	exercise, blood glucose monitoring, foot care, and smoking [41].
Medication adherence	This will be measured with the 6-item validated self-reported Brooks Medication Adherence Scale (BMAS) [42].
Depression	The Patient Health Questionnaire (PHQ)-9 is a brief questionnaire that scores each of the nine DSM-IV criteria for depression [43].
Anxiety	General anxiety disorder (GAD) will be assessed using the 7-item anxiety scale [44].
Covariates	
Sociodemographics	Previously validated items from the National Health Interview Survey will be used to capture demographic characteristics including age, sex, race/ethnicity, marital status, education, employment, income, and insurance [45].
Health literacy	A 3-item literacy scale noting capacity to obtain, process, and understand basic health-related decisions will measure health literacy [46].
Comorbidities	Previously validated items from the Behavioral Risk Factor Surveillance System will be used to assess the presence of comorbidities [47].

**Table 3:** Study timeline.

Year	1				2				3			
Quarters	1	2	3	4	1	2	3	4	1	2	3	4
**Start-up Activities**												
Obtain IRB approval												
Register trial on clinicaltrials.gov												
Participant recruitment and enrollment												
30 participants enrolled												
70 participants enrolled												
100 participants enrolled												
Intervention delivery												
Data analysis												
Larger grant submission and final reports												
Manuscripts												

## Data Availability

Data sharing is not applicable. No new data were created or analyzed in this study.
